# Membrane-bound β-catenin degradation is enhanced by ETS2-mediated Siah1 induction in *Helicobacter pylori*-infected gastric cancer cells

**DOI:** 10.1038/oncsis.2017.26

**Published:** 2017-05-08

**Authors:** L Das, S B Kokate, P Dixit, S Rath, N Rout, S P Singh, S E Crowe, A Bhattacharyya

**Affiliations:** 1 School of Biological Sciences, National Institute of Science Education and Research (NISER) Bhubaneswar, Jatni, Odisha, India; 2 Department of Oncopathology, Acharya Harihar Regional Cancer Centre, Cuttack, Odisha, India; 3 Department of Gastroenterology, SCB Medical College, Cuttack, Odisha, India; 4 School of Medicine, Division of Gastroenterology, UC San Diego, California, USA

## Abstract

β-catenin has two different cellular functions: intercellular adhesion and transcriptional activity. The E3 ubiquitin ligase Siah1 causes ubiquitin-mediated degradation of the cytosolic β-catenin and therefore, impairs nuclear translocation and oncogenic function of β-catenin. However, the effect of Siah1 on the cell membrane bound β-catenin has not been studied. In this study, we identified that the carcinogenic bacterium *H. pylori* increased ETS2 transcription factor-mediated Siah1 protein expression in gastric cancer cells (GCCs) MKN45, AGS and Kato III. Siah1 protein level was also noticeably higher in gastric adenocarcinoma biopsy samples as compared to non-cancerous gastric epithelia. Siah1 knockdown significantly decreased invasiveness and migration of *H. pylori*-infected GCCs. Although, Siah1 could not increase degradation of the cytosolic β-catenin and its nuclear translocation, it enhanced degradation of the membrane-bound β-catenin in the infected GCCs. This loss of membrane-bound pool of β-catenin was not associated with the proteasomal degradation of E-cadherin. Thus, this work delineated the role of Siah1 in increasing invasiveness of *H. pylori*-infected GCCs.

## Introduction

Siah family of E3 ubiquitin ligases are involved in the proteasome-dependent degradation of target proteins. These proteins modulate several cellular functions including angiogenesis, inflammation, cell proliferation, cell migration and apoptosis.^1, 2, 3, 4, 5^ Humans have *siah1*, *siah2* and *siah3* genes whereas, mice have *siah1a*, *siah1b* and *siah2* genes.^6, 7^ Animal model-based studies support oncogenic functions of both Siah1 and Siah2 while tumor-promoting roles of Siah2 and apoptosis-inducing role of Siah1 are mostly reported from cell line-based assays.^8^

Siah1 is involved in the p53-mediated degradation of the oncogene β-catenin in the cytosol.^9, 10, 11^ β-catenin is involved in the cell–cell adhesion, as well as Wnt-signaling in epithelial cells.^12, 13^ Two separate pools of β-catenin maintain these two very different functions—a nonmembranous cytoplasmic-nuclear pool and a cell membrane pool bound with E-cadherin.^14^ In the absence of Wnt-signaling, the cytoplasmic β-catenin is degraded by the ubiquitin-proteasomal degradation pathway. This results in the accumulation and translocation of β-catenin to the nucleus and causes transcriptional activation of its target genes.^15^

Siah1-mediated degradation of cytoplasmic β-catenin is a phosphorylation-independent mechanism. While several studies reported about proteasomal degradation of β-catenin in the cytosolic compartment, very little is known about its proteasomal degradation in the cell membrane adherens junction. However, this is clear that cell membrane-bound β-catenin degradation is associated with the loosening of the cell–cell attachment. The E3 ubiquitin ligase Hakai ubiquitinates cell membrane-bound E-cadherin and β-catenin leading to the internalization of the E-cadherin complex and enhanced epithelial cell migration.^16, 17^ What happens to the cadherin-bound β-catenin after the complex is dissociated from the adherens junction is not clearly known but it is considered to be either degraded or recycled.^18^ Another E3 ubiquitin ligase, Ozz-E3, ubiquitinates β-catenin and causes its proteasomal degradation.^19^ Since there is only one report of a rare inactivating mutation of *siah1* in gastric cancer^20^ and none in any other cancers,^21^ the cellular function of Siah1 in regulating gastric cancer possibly is not limited to its tumor-suppressive role. This notion is further supported by animal studies, which have shown Siah1 as a tumor-promoter.^8^

We sought to investigate the effect of *H. pylori* infection on the expression and activity of Siah1 protein in the infected gastric cancer cells (GCCs). Our findings reveal a novel mechanism of *H. pylori* pathogenesis wherein *H. pylori*-mediated upregulation of ETS2 induces *siah1* transcription. We observe that Siah1 causes membrane-bound β-catenin degradation and induces invasiveness and migration of infected GCCs.

## Results

### Siah1 mRNA and protein levels in *H. pylori*-infected human GCCs

**Figure 1:**
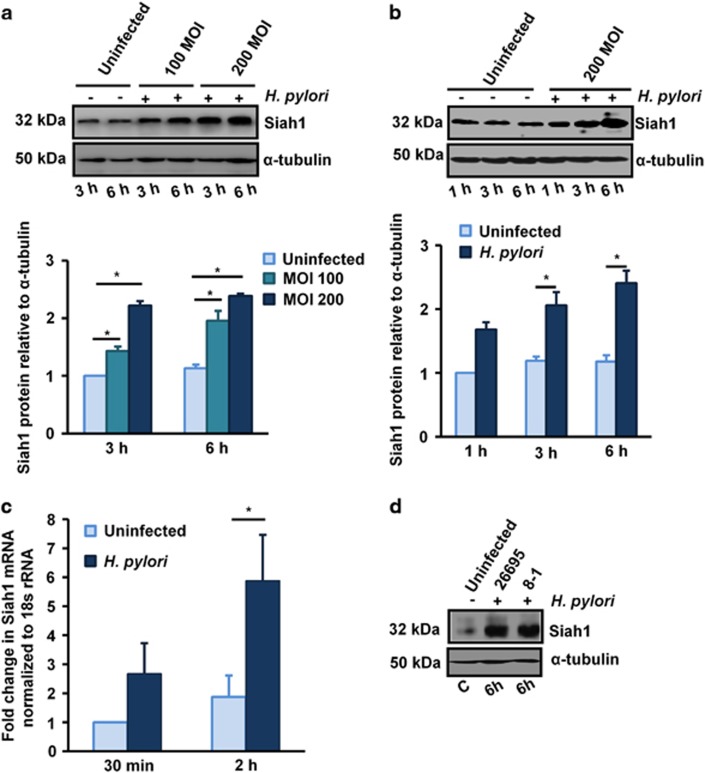
*H. pylori* enhance Siah1 expression in GCCs. (**a**) Western blottings of whole cell lysates from uninfected and *H. pylori*-infected (3 h and 6 h with MOI 100 and 200) MKN45 cells show increased Siah1 protein levels in infected cell lysates. α-tubulin is the loading control. (**b**) Time kinetics of Siah1 protein induction (1–6 h) in *H. pylori*-infected MKN45 cells. (**c**) Real time RT-PCR shows increased Siah1 mRNA expression in *H. pylori*-infected MKN45 cells. Bars shown in panels A–C represent normalized data (mean±s.e.m., *n*=3), *P<0.05. (**d**) Western blot results of showing Siah1 protein levels from cell lysates isolated from uninfected and *H. pylori*-infected MKN45 cells. Two different strains of *H. pylori* compared are *cag* PAI(+) strain 26695 and *cag* PAI(-) strain 8-1.

To study the Siah1 protein level in *H. pylori*-infected gastric cancer cells (GCCs), MKN45 cells were infected with a cytotoxin-associated gene pathogenicity island-positive {*cag* PAI(+)} *H. pylori* strain 26695 at a multiplicity of infection (MOI) 100 and 200 for 3 h and 6 h. Western blot analysis revealed that although MOI 100 at 6 h post infection (p.i.) and MOI 200 at both 3 h and 6 h significantly induced Siah1 protein, MOI 200 at 6 h was maximally effective (Figure 1a). To identify the optimal time for Siah1 protein induction with 200 MOI of *H. pylori* infection, MKN45 cells were infected for 1 h, 3 h and 6 h. Representative western blot results (*n*=3) showed that 6 h was the optimal time required for Siah1 protein induction (Figure 1b). To assess the effect of *H. pylori* infection on Siah1 transcription, MKN45 cells were infected with MOI 200 of *H. pylori*. The real-time RT-PCR data (*n*=3) confirmed that Siah1 messenger RNA (mRNA) was significantly (*P <0.05) enhanced after 2 h p.i. as compared to the uninfected control (Figure 1c). Comparison of the *cag* PAI(+) strain with an isogenic *cag* PAI(-) mutant strain 8-1 revealed that Siah1 protein induction was a *cag* PAI-independent event (Figure 1d). So, all future experiments used only strain 26695, unless specified differently.

### ETS2 binds with siah1 5′ UTR and enhances Siah1 transcription in the *H. pylori*-infected GCCs

**Figure 2:**
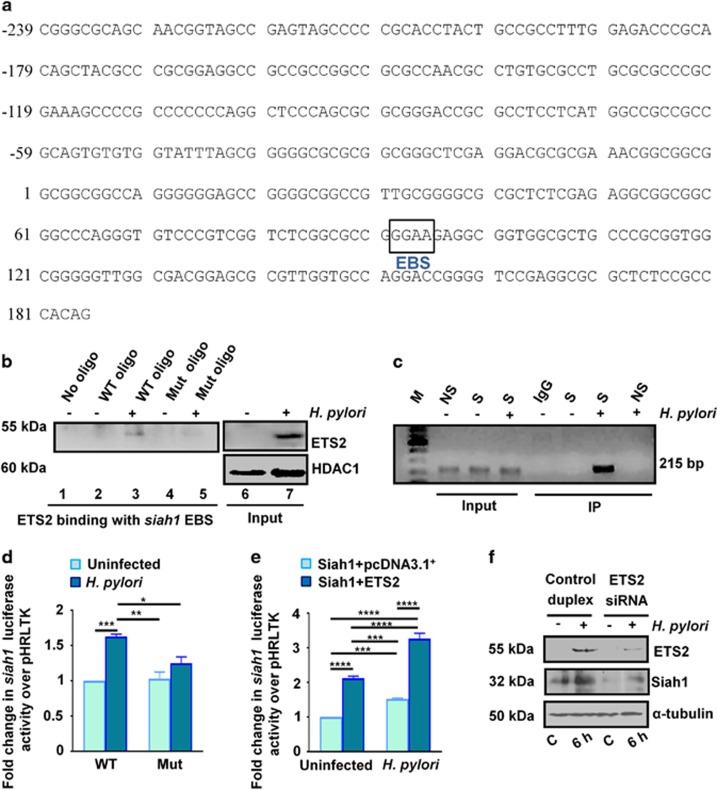
ETS2 binds to EBS in the 5′ UTR and induces *siah1* transcription and protein expression in the *H. pylori*-infected GCCs. (**a**) Promoter and 5′ UTR analysis of human *siah1* gene shows that an EBS located between +92 and +95 (represented by a box). We assume that the most upstream exon 1 of the Siah1 cDNA is at position +1^22^. (**b**) Western blot results showing the status of ETS2 binding with the *siah1* 5′ UTR (*n*=3) in the presence or absence of *H. pylori*. ETS2 binds to the WT EBS only but not with the EBS-Mut oligo. Western blot of nuclear lysates shows the levels of ETS2 protein expression in the input lanes. HDAC1 is the loading control for nuclear lysates. (**c**) ChIP assay of ETS2 immunocomplex for *siah1* EBS. IgG= immunoglobulin G; M= MW marker; NS= non-specific primer, S= specific primer. (**d**) Figure shows dual luciferase assay involving WT and ETS2-Mut *siah1* 5′ UTR-transfected and infected or uninfected MKN45 cells. Data are analyzed by two-way ANOVA with Tukey’s post hoc test (*n*=3). Error Bars, s.e.m. ***P< 0.001, **P< 0.01, *P< 0.05. (**e**) Bar graph of dual luciferase assay result showing transcriptional activation of WT *siah1* 5’ UTR with ectopic *ETS2* expression and *H. pylori* infection. Data are analyzed by two-way ANOVA with Tukey’spost hoc test. Error bars, s.e.m. ***P<0.003; ****P< 0.0001. (**f**) Transient transfection of ETS2 siRNA followed by western blotting shows Siah1 suppression in the ETS2-suppressed MKN45 cells.

To identify the transcription factor responsible for Siah1 upregulation in the *H. pylori*-infected human GCCs, *siah1* promoter and 5′ UTR analysis was done using the Genomatix Suite of sequence analysis tool MatInspector (professional version 6.2.2). We identified the presence of an E26 transformation-specific (ETS) binding site (EBS) in the *siah1* 5′ UTR (core element GGAA located between +92 and +95, GenBank: AJ400626.1)^22^ (Figure 2a).

*In vitro* binding assay was performed to study ETS2 binding with the EBS of the 5′ UTR of *siah1* in uninfected and infected MKN45 cells. WT and EBS-mut oligonucleotides were 5′ biotinylated (Supplementary Figure 1). Nuclear extracts prepared from uninfected or 3 h *H. pylori*-infected MKN45 cells were incubated with biotinylated-oligonucleotides coated over magnetic beads. Western blots of bead-bound proteins showed ETS2 binding with the *siah1* promoter EBS only in the infected cells (Figure 2b). Western blot of input nuclear lysates showed ETS2 protein level in the nuclear fraction. HDAC1 was used as a nuclear loading control. *In vivo* binding of ETS2 with *siah1* EBS was further confirmed by chromatin immunoprecipitation (ChIP) assay. Uninfected and *H. pylori*-infected MKN45 cells were immunoprecipitated using ETS2 specific antibody. PCR products obtained from the immunocomplex represented the *siah1* promoter and 5′ UTR flanking the EBS (S=specific PCR product). PCR product was not obtained from the 5′ far upstream sequence (NS=non-specific PCR product) (Figure 2c).

Next, dual luciferase assays were performed to study the effect of ETS2 on *siah1* transactivation. WT or EBS-Mut *siah1* reporter constructs were co-transfected with *Renilla* luciferase construct phRLTK in MKN45 cells followed by infection with *H. pylori* for 1 h. Data confirmed that *H. pylori* significantly increased *siah1* transactivation in the WT *siah1* EBS-expressing cells as compared to the mut EBS-expressing cells (Figure 2d). Reduced *siah1* transactivation in EBS-mut *H. pylori*-infected MKN45 cells compared with WT EBS-expressing *H. pylori*-infected cells further confirmed the positive effect of ETS2 on *siah1* transcription in *H. pylori-*infected GCCs. Further, dual luciferase assay was performed with co-transfection of empty vector or ETS2 overexpression plasmid along with the WT *siah1* promoter construct and the *Renilla* luciferase construct phRLTK followed by infection for 1 h. Results (Figure 2e) confirmed that ETS2 could significantly enhance *H. pylori*-mediated *siah1* transcription.

To find out the role of *ETS2* knockdown on Siah1 protein level, we transfected MKN45 cells with siETS2 or control duplex and infected with *H. pylori or* left uninfected. A noticeable decrease in *H. pylori*-induced Siah1 protein level was observed in ETS2-suppressed cells after *H. pylori* infection as compared to the control duplex-transfected cells. This confirmed the role of ETS2 in inducing Siah1 protein during *H. pylori* infection (Figure 2f).

**Figure 3:**
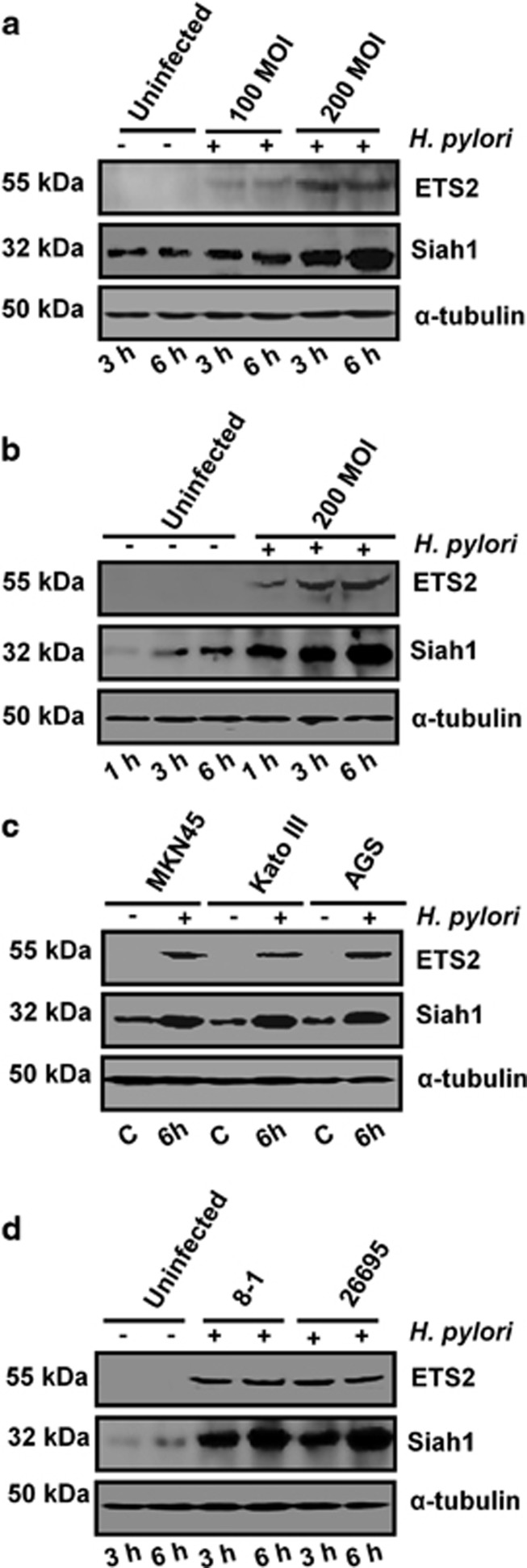
Parallel ETS2 and Siah1 induction occurs in *H. pylori-*infected GCCs, as well as in human gastric adenocarcinoma biopsy samples. (**a**) A representative western blot (*n*=3) showing optimal induction of ETS2 and Siah1 by 200 MOI of *H. pylori* at 3 h and 6 h p.i. (**b**) Cells infected for various time periods are analyzed for ETS2 and Siah1 protein expression (*n*=3). (**c**) Western blot (*n*=4 showing ETS2 and Siah1 proteins in uninfected and *H. pylori*-infected Kato III, MKN45 and AGS cells. (**d**) Western blot results (*n*=3) depicting equal effectiveness of 8-1 and 26695 in inducing ETS2 and Siah1 proteins. Graphical representations of panels A–D are shown in Supplementary Figure 2.

ETS transcription factors are crucial for cancer progression.^23^ We also studied the status of ETS2 along with Siah1 proteins after infection with 100 and 200 MOI at 3 and 6 h. *H. pylori* MOI and time-dependently increased ETS2 as well as Siah1 proteins in MKN45 cells and 200 MOI was optimal (Figure 3a). To find out the expression of Siah1 and ETS2 at 200 MOI, MKN45 cells were infected for 1, 3 and 6 h. Although ETS2 and Siah1 expressed from 1 h p.i., 3 and 6 h infection resulted in highly-induced expression of both of these proteins (Figure 3b). Like MKN45, other GCCs, that is, Kato III and AGS also showed ETS2 and Siah1 expression at 6 h of infection with MOI 200 (Figure 3c). Siah1 was earlier reported to be activated by p53.^9, 22, 24^ As Kato III cells are p53-null cells, our data confirmed that Siah1 expression in *H. pylori*-infected GCCs was p53-independent. In addition, 26695 and 8-1 strains equally induced ETS2 and Siah1 proteins in MKN45 cells (Figure 3d) suggesting of *cag* PAI-independent regulation of Siah1. Figures 3a–d are graphically presented in Supplementary Figure 2.

### *Helicobacter*-infected human and mouse gastric epithelia show enhanced expression of ETS2 and Siah1

**Figure 4:**
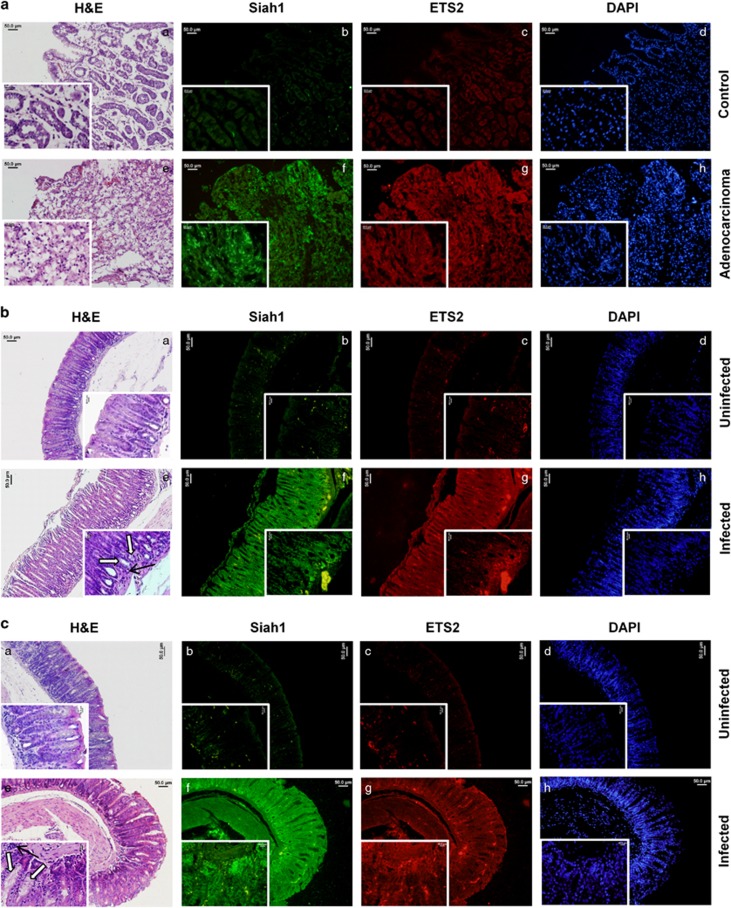
Induced ETS2 and Siah1 expression in *Helicobacter*-infected human and mouse gastric epithelia. (**a**) H&E staining of human non-cancer (a) and adenocarcinoma (e) biopsy samples (*n*=10 for each group) and fluorescence microscopy of the staining for Siah1 (b and f), ETS2 (c and g) and DAPI (d and h). Original magnification × 100, inset × 400. Scales shown 50 μm. Inset scale 20 μm. (**b**) H&E staining of uninfected (*n*=16) and infected (*n*=16) antral gastric tissues from C57BL/6 mice (a and e, respectively) and their corresponding fluorescence microscopy images showing Siah1 (b and f), ETS2 (c and g) and DAPI (d and h) staining. Infected mice show inflammation (thin arrow), mucus gland metaplasia (open arrow) in the mucosa. (**c**) Data representing similar observations in another set of uninfected and infected mice gastric tissues. Original magnification × 100, inset × 400. Scales shown in **b** and **c**: 50 μm.

ETS2 and Siah1 were assessed in gastric adenocarcinoma, antral biopsy samples (stage III, rapid urease test-positive) collected from consenting patients. H&E staining and fluorescence microscopy showed marked induction of ETS2 and Siah1 in adenocarcinoma samples (*n*=10) compared to non-cancer tissues (*n*=10) (Figure 4a). These data showed coexistence of ETS2 and Siah1 proteins in *H. pylori*-mediated gastric adenocarcinoma.

C57BL/6 mice were infected with *H. felis* as this model represents the classical cascade of *H. pylori*-driven carcinomatous changes observed in humans.^25^ Eight months p.i. all infected mice showed precancerous lesions represented by marked mucus-gland metaplasia, enhanced ETS2 and Siah1 expression (*n*=16) as compared to the uninfected tissues (*n*=16) (Figures 4b and c).

### Siah1 enhances loss of membrane-bound β-catenin in the *H. pylori*-infected GCCs

**Figure 5:**
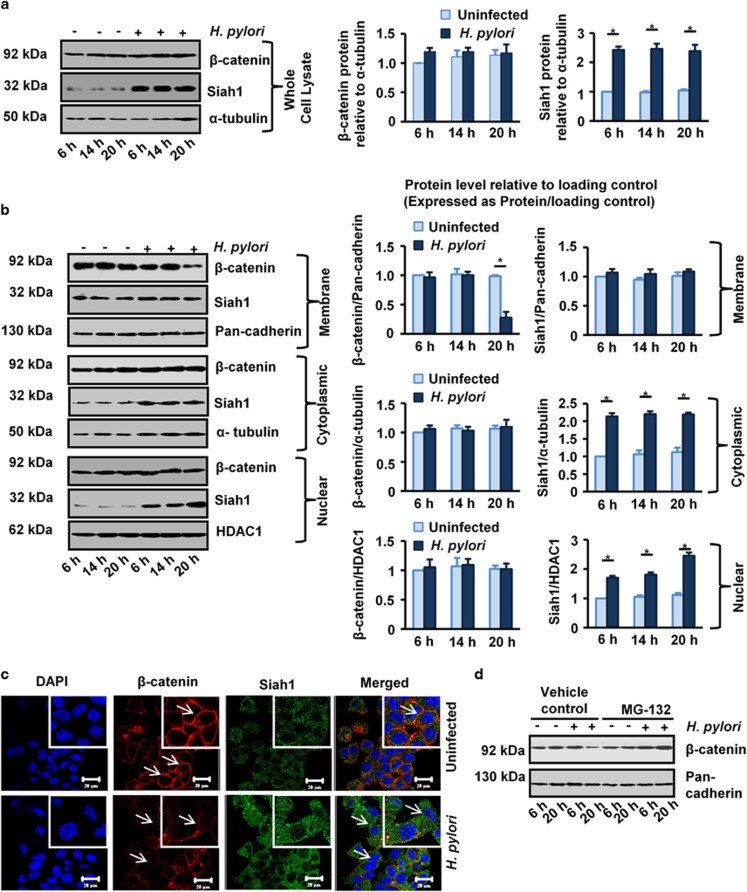
Membrane-bound β-catenin degradation in *H. pylori*-infected GCCs is proteasome-mediated. (**a**) Western blotting for Siah1 and β-catenin in uninfected and *H. pylori*-infected (6 h, 14 h and 20 h) MKN45 whole cell lysates (*n*=3). (**b**) Western blotting of membrane, cytoplasmic and nuclear fractions of uninfected or *H. pylori*-infected lysates for detection of Siah1 and β-catenin proteins (*n*=3). Pan-cadherin, α-tubulin and HDAC1 are used as respective loading controls. Bars depicting panels A–B represent normalized data (mean±s.e.m., *n*=3), *P<0.05. (**c**) A representative confocal microscopy result (*n*=3) showing degradation of membrane-bound β-catenin in *H. pylori-*infected MKN45 cells. (**d**) Western blot (*n*=3) showing rescue of membrane-bound β-catenin from degradation by treatment with the proteasome inhibitor, MG132.

Siah1 was previously reported to induce apoptosis and cell-cycle arrest by degradation of cytosolic β-catenin.^9, 10^ Siah1 enhanced β-catenin degradation in the cytosolic compartment of hepatocytes and thereby reduced its nuclear localization and effectiveness as an oncogenic factor.^26^ The same study, however, showed that the membrane-bound β-catenin was not affected by Siah1. Siah1 was also reported to degrade cytosolic β-catenin in the cervical epithelial cancer cells.^27^ To find out the effect of *H. pylori* infection on β-catenin, we performed western blot of whole cell lysates prepared from *H. pylori*-infected (6, 14 and 20 h) or uninfected MKN45 cells. Data indicated that although Siah1 was significantly (*P<0.05) induced by *H. pylori*, β-catenin level in the whole cell lysate did not change with *H. pylori* infection (Figure 5a). We next assessed the membrane-enriched, cytoplasmic and nuclear-fraction-enriched lysates by western blotting. Pan-cadherin, α-tubulin and histone-deacetylase 1 (HDAC1) served as loading controls for the membrane fractions, cytoplasmic fractions and nuclear fractions, respectively. Membranous β-catenin was significantly (*P< 0.05) downregulated only after 20 h of *H. pylori* infection (Figure 5b). However, no change in membrane-bound Siah1 was noted following *H. pylori* infection. Surprisingly, cytoplasmic fractions which showed significantly high (*P< 0.05) Siah1 protein levels after *H. pylori* infection at all time points, did not demonstrate any loss of β-catenin in infected cells. P.i., nuclear fractions also showed a time-dependent significant (*P< 0.05) increase in Siah1 but unchanged β-catenin protein level. Confocal microscopy performed on MKN45 cells further confirmed that expression of only the membrane-bound β-catenin was highly reduced 20 h p.i. (Figure 5c). To find out whether membrane β-catenin loss was a proteasome-dependent degradation process or not, we infected MKN45 cells with *H. pylori* in the presence or absence of 50 μM MG132, a proteasome inhibitor. *H. pylori*-mediated downregulation of membrane-bound β-catenin was markedly rescued by MG132 treatment indicating that *H. pylori*-mediated membrane-bound β-catenin loss was due to proteasomal degradation (Figure 5d).

**Figure 6:**
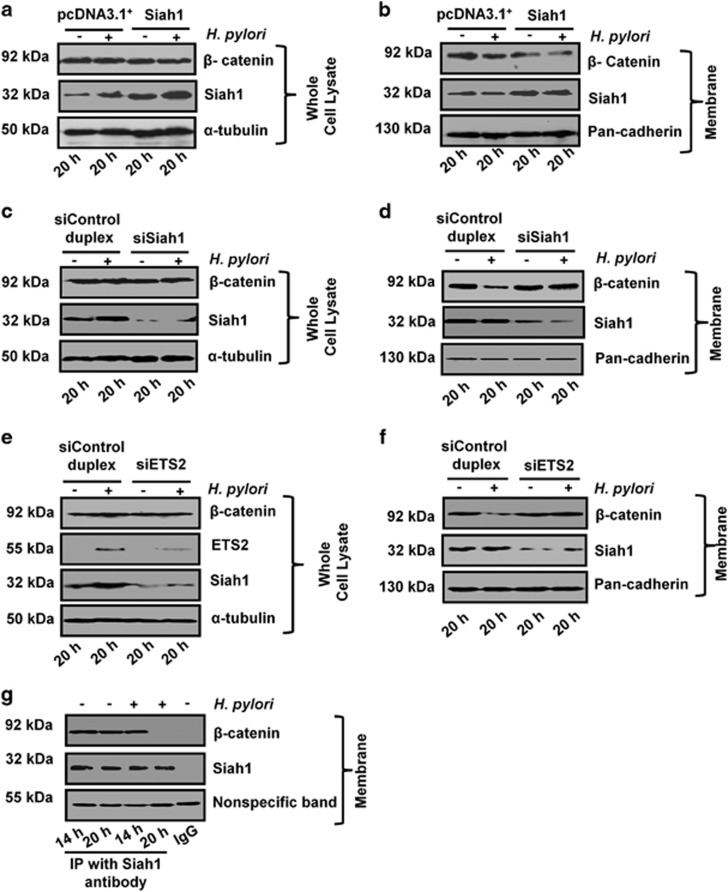
Siah1 promotes membrane-bound β-catenin degradation in *H. pylori*-infected GCCs. (**a**) Western blotting result (*n*=3) of whole cell lysates prepared from Siah1 or empty vector (pcDNA3.1^+^)-transfected and *H. pylori*-infected or uninfected MKN45 cells. Blots are incubated with Siah1 and β-catenin primary antibodies. α-tubulin is the loading control. (**b**) Decreased membrane-bound β-catenin expression is detected by western blotting (*n*=3) in Siah1-overexpressed and *H. pylori*-infected cells. (**c**) Western blotting result (*n*=3) of whole cell lysates prepared from siSiah1 or siControl duplex-transfected and *H. pylori*-infected or uninfected MKN45 cells. Blots are incubated with Siah1 and β-catenin primary antibodies. α-tubulin is the loading control. (**d**) Decreased membrane-bound β-catenin expression is seen in Siah1-supressed and *H. pylori*-infected cells. (**e**) Western blotting result (*n*=3) of whole cell lysates prepared from siETS2 or siControl duplex-transfected and *H. pylori*-infected MKN45 cells. Blots are incubated with Siah1 and β-catenin primary antibodies. α-tubulin is the loading control. (**f**) Decreased membrane-bound β-catenin expression is detected in western blotting (*n*=3) in ETS2-supressed and *H. pylori*-infected cells. (**g**) Siah1 is immunoprecipitated with anti-Siah1 antibody from the membrane fraction and immunoblotted to detect β-catenin and Siah1 interaction. Non-specific band=immunoglobulin heavy chain. Graphical representations are shown in Supplementary Figure 3.

To assess the effect of *siah1*and *ETS2* on membrane β-catenin level, we performed a few assays. Siah1-overexpressed MKN45 cells showed no change in β-catenin in whole cell lysates even after 20 h of *H. pylori* infection (Figure 6a). With Siah1 overexpression, however, β-catenin loss in the membrane was significantly enhanced (Figure 6b). siSiah1 transfection had no impact on the β-catenin level in whole cell lysates (Figure 6c). However, siSiah1 transfection significantly (*P<0.05) blocked *H. pylori*-mediated membrane β-catenin degradation (Figure 6d). Next, we tried to assess the effect of *ETS2* knockdown on Siah1 protein. siETS2 significantly (*P<0.05) reduced ETS2, as well as Siah1 protein levels in the whole cell lysates (Figure 6e). Post siETS2 transfection, cell membrane fractions showed significant reduction in membrane Siah1 level (Figure 6f). siETS2 could significantly (*P< 0.05) decrease *H. pylori*-driven membrane β-catenin degradation. Data shown in Figures 6a and f have been graphically presented in Supplementary Figure 3. Interaction of Siah1 with membrane-bound β-catenin was studied by immunoprecipitation assay of membrane-rich lysates. Result (Figure 6g) showed that although Siah1 interacted with β-catenin in uninfected or infected cells at 14 h and in uninfected cells at 20 h, β-catenin was completely lost in the immunoprecipitate only after 20 h of infection.

As both E-cadherin and β-catenin are degraded by E3 ubiquitin ligases^16^ and previous studies reported about loss of E-cadherin from the membrane in *H. pylori*-infected GCCs,^28, 29^ we wanted to assess E-cadherin status in the membrane of Siah1-overexpressed cells in the presence or absence of *H. pylori*. For this, MKN45 cells were transfected with the empty vector (pcDNA3.1^+^) or Siah1 construct. Transfected cells were infected with *H. pylori* for 20 h in the presence or absence of either vehicle control or 50 μM MG132. Data revealed that neither Siah1 overexpression nor *H. pylori* promoted membrane-bound E-cadherin loss in GCCs (Supplementary Figure 4). Western blotting of the membrane-fraction showed E-cadherin expression under the above experimental conditions.

### Siah1 promotes migration and invasiveness of *H. pylori*-infected GCCs

**Figure 7:**
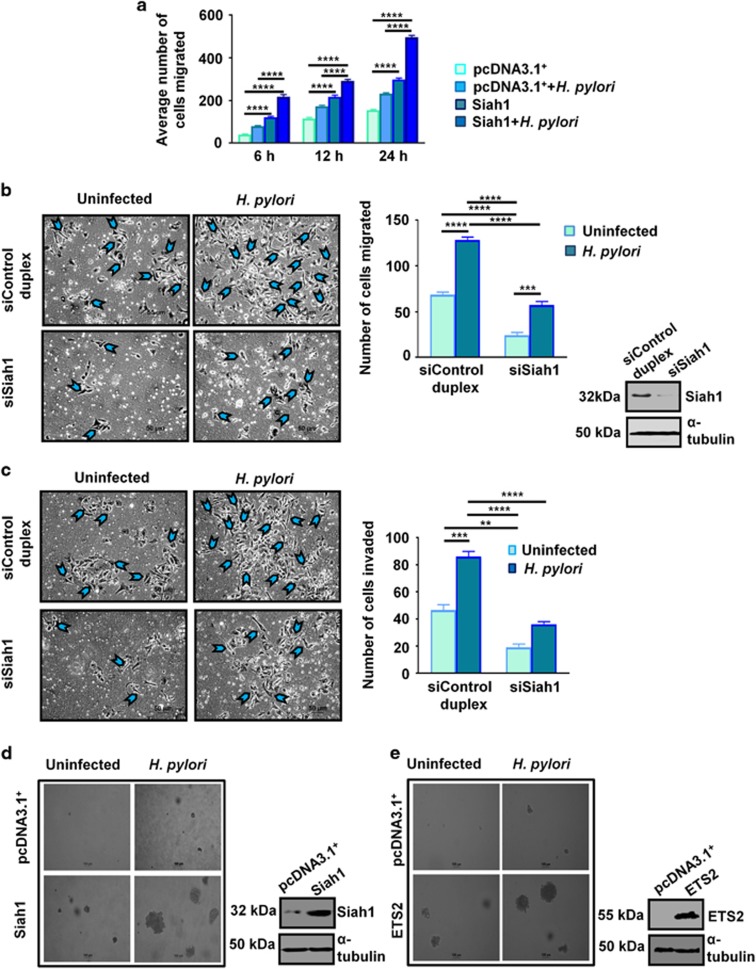
Siah1 increases the rate of cell migration in *H. pylori*-infected GCCs. (**a**) Graphical representations (*n*=3) of wound healing assay showing enhanced migration potential of Siah1-overexpressed and *H. pylori*-infected cells. 24 h post transfection, wound is marked and the scratched area is monitored from 6 h-24 h. Data have been analyzed by 2-way ANOVA with Tukey’s post hoc test. Error bars, s.e.m. ****P<0.0001. (**b**) Cell migration assay performed in transwell chambers with AGS cells show decreased migration of infected cells expressing siSiah1 as compared to the siControl group. Arrowheads indicate migrated cells; scales shown: 50 μm. Bar graphs denote the average number of migrated cells (mean±s.e.m., *n*=3). Data are analyzed by 2-way ANOVA with Tukey’s post hoc test. Error bars, s.e.m. ***P<0.003; ****P<0.0001. Protein level of siSiah1 cells are shown in the accompanying western blot images. (**c**) Matrigel invasion assay with Siah1-suppressed AGS cells showing reduced invasiveness in *H. pylori*-infected GCCs. Bar graphs denote the average number of cells invaded through the Transwell matrigel (*n*=3). Arrowheads indicate invaded cells. Data are analyzed by two-way ANOVA with Tukey’s post hoc test. Error bars, s.e.m. **P<0.01; ***P<0.003; ****P<0.0001. Scales shown: 50 μm. (**d**) Soft agar colony formation assay is performed on MKN45 cells. Siah1 stably-transfected cells show a substantial increase in colony forming ability post *H. pylori* infection as compared to cells expressing the empty vector. Siah1 level in Siah1-stable cells are shown in the accompanying western blot result. (**e**) Soft agar assay performed with uninfected or infected MKN45 cells that stably-express either ETS2 or empty vector show a substantial increase in colony forming ability of ETS2-expressing cells. Protein level of ETS2 stable cells are shown in the accompanying western blot images. Scales shown in **b** and **c**: 100 μm.

It is well established that loss of membrane-bound β-catenin enhances cancer invasiveness.^30, 31, 32^ Membrane-bound β-catenin loss is also frequent in metastatic gastric cancer cases.^33^ As we found that Siah1 could increase membrane-bound β-catenin degradation and it is also well known that *H. pylori* induces invasiveness of infected cells,^34^ we next wanted to assess the effect of Siah1 in increasing metastatic properties in GCCs. To evaluate the influence of Siah1 overexpression on the ability of migration of *H. pylori*-infected GCCs, we performed wound-healing assay. For this, the highly adherent gastric adenocarcinoma cell AGS was preferred over the partially adherent MKN45 cells (poorly-differentiated gastric adenocarcinoma cells) since MKN45 cells partly grow in clumps.^35^ pcDNA3.1^+^ or Siah1 stably-expressing AGS cells were grown in monolayer, a wound was marked and cells were incubated in the presence or absence of *H. pylori* for various time periods. A significant time-dependent increase in cell migration was observed in Siah1-expressing *H. pylori*-infected AGS cells than empty-vector-expressing infected cells (Figure 7a). To further confirm the role of Siah1 in cell migration, Transwell migration assay was performed with Siah1-suppressed AGS cells. siSiah1-expressing cells showed significantly less *H. pylori*-induced cell migration (Figure 7b). Cell invasiveness was studied by Matrigel-invasion assay using exogenous siSiah1-expressing AGS cells. Significantly less (*P< 0.05) cell invasion was observed in siSiah1-expressing infected AGS cells as compared to control duplex-expressing infected AGS cells (Figure 7c). Western blot results alongside the Transwell migration assay data indicated status of siSiah1. Soft agar colony formation assay was performed to study anchorage-independent growth of *H. pylori-*infected cells. Both Siah1 or ETS2-expressing AGS cells showed markedly increased colony formation in soft agar plates as compared to empty vector-expressing *H. pylori-*infected cells (Figure 7d and e, respectively). Western blot results alongside the soft agar assay data indicated status of Siah1 and ETS2 proteins in the respective stable cells used for soft agar assays.

## Discussion

*H. pylori* colonization enhances inflammatory responses and neoplastic changes owing to the loss of the gastric epithelial cell barrier function. Loss of cell-to-cell adhesions takes place in the infected epithelium as disruption of the junctional cadherin-catenin complex occurs. Here, we report for the first time that ETS2 enhances *siah1* transcription in the *H. pylori*-infected GCCs. We prove that *H. pylori*-mediated Siah1 upregulation promotes invasiveness of *H. pylori*-infected GCCs by increasing degradation of the membrane-bound β-catenin. In addition, we also show that increased expression of Siah1 protein in *H. pylori*-infected GCCs does not cause E-cadherin loss from the cell membrane.

Cadherin-bound β-catenin is an integral component of the adherens junctions. An E3 ubiquitin ligase Hakai induces E-cadherin ubiquitination and internalization by endocytosis.^16^ Endocytosis possibly plays a role in releasing β-catenin from the internalized E-cadherin complex.^36^ Otherwise, tyrosine phosphorylation of β-catenin can also disrupt the α-catenin-E-cadherin association.^37^ It is believed that E-cadherin-bound β-catenin can accumulate at the perinuclear endocytic recycling compartment and translocate to the nucleus upon Wnt activation.^38^ After internalization, Hakai-mediated E-cadherin ubiquitination redirects the latter from a recycling pathway to a lysosome-mediated degradation process.^39^ Membrane-bound β-catenin is also targeted for ubiquitin-mediated degradation by E3 ubiquitin ligases Hakai and Ozz.^16, 19^ Other ubiquitin ligases such as MDM2 and K5, can also ubiquitinate and degrade E-cadherin.^40, 41^ We show here that the E3 ubiquitin ligase Siah1 enhances degradation of the cell membrane-bound β-catenin in the *H. pylori*-infected GCCs which can be prevented by inhibiting proteasomal degradation.

This study reveals that Siah1 mediated membrane β-catenin degradation is independent of membrane E-cadherin status. Interestingly, *H. pylori* CagA can interact with E-cadherin and to disrupt the E-cadherin-β-catenin complex. Thereby, CagA increases cytoplasmic β-catenin degradation and its nuclear accumulation.^42^ In contrast to this, we notice no β-catenin loss in the cytosol and no nuclear β-catenin accumulation in MKN45 cells by the *cag* PAI(+) *H. pylori* strain. Our findings corroborate another report by Bebb *et al.* which shows that the reduction in membrane β-catenin occurs in *H. pylori*-infected cells without concomitant increase in its cytosolic and nuclear pool.^43^ However, as only nonphosphorylated CagA interacts with E-cadherin^42, 44^ while CagA gets phosphorylated in the infected host,^45, 46^ cell disruption of E-cadherin-β-catenin interaction by CagA in our system is ruled out. We note that loss of membrane-bound β-catenin only takes place in *H. pylori*-infected GCCs but not in uninfected GCCs. This could be due to increased activity of Siah1 protein in *H. pylori*-infected GCCs. Research is currently underway in our laboratory to identify the possible role of posttranslational modification(s) in regulating Siah1 activity and whether that is restricted to the cell membrane.

Siah1-mediated cytosolic β-catenin degradation is thought to have an overall tumor-suppressive effect. We find that Siah1 protein is induced by ETS2 in *H. pylori*-infected GCCs but it does not cause any change in the cytosolic and nuclear pool of β-catenin. These observations are in complete contradiction with earlier findings which have shown that the cytosolic fraction of β-catenin is degraded by Siah1 and thus, Siah1 act as a tumor suppressor.^9, 10, 47^ As Siah1 can be upregulated in a p53-dependent manner,^20, 48, 49^ association of Siah1 with apoptosis is also widely accepted. Surprisingly, we find that the level of Siah1 protein in *H. pylori*-infected GCCs is not dependent on p53 as it is also induced in the p53-null Kato III cells. As basal expression of Siah1 is noticed in uninfected GCCs when ETS2 protein is not expressed, it is clear that p53 and ETS2-independent mechanisms of *siah1* transcription exist.

E3 ubiquitin ligases are involved in various cellular processes including cancer and inflammation.^3, 50^ Recently we have shown that Siah2 was induced by ETS2 and Twist1 in *H. pylori*-infected GCCs.^51^ It is therefore evident from the current study that ETS2 can simultaneously induce both Siah1 and Siah2 proteins in *H. pylori*-infected GCCs. As Siah1 lacks Twist1-binding site, we speculate that these isotypes can still be differentially regulated since ETS2 and Twist1 expression might vary depending on the staging of gastric cancer. As we have not observed any role of Siah2 in membrane-bound β-catenin degradation in *H. pylori*-infected GCCs (unpublished data), target molecules of these Siah isotypes might very well be different. We believe that despite having a common transcription factor ETS2, Siah1 and Siah2 have unique roles in the cell and disease processes.

Since this work uncovers a new mechanism of *H. pylori*-induced degradation of cell membrane-associated β-catenin, we believe that Siah1 is a crucial factor regulating gastric cancer progression and metastasis. Stage-dependent variation in Siah1-β-catenin interaction might exist that can determine the course of treatment-outcome. For this, further studies with patient samples are required which will surely enrich our current knowledge of the molecular basis of *H. pylori*-mediated gastric cancer pathogenesis.

## Materials and methods

### Cells, plasmids, siRNAs and inhibitors

The human adenocarcinoma GCCs MKN45, Kato III, AGS were cultured and maintained, as described earlier.^52, 53^ Full-length wild type (WT) human Siah1 cDNA was subcloned in HindIII/XhoI restriction sites of eukaryotic expression vector pcDNA3.1^+^ (Invitrogen, CA, USA). ETS2 construct (#28128) was procured from Addgene, MA, USA. The full length human *siah1* promoter was cloned into the pGL3 basic vector (Promega, WI, USA) using restriction sites KpnI and HindIII. The promoter construct s*iah1* WT was used as a template to generate mutation at the ETS2-binding site (EBS) using Quik Change site-directed mutagenesis kit (Agilent Technologies, CA, USA). Constructs were confirmed by sequencing. Primer sequences are shown in Supplementary Figure 1. sisiah1 were purchased from Santa Cruz Biotechnology, Texas, USA, whereas siETS2 was purchased from Origene, MD, USA. The proteasome inhibitor Z-Leu-Leu-Leu-al (MG132) was purchased from Sigma-Aldrich, WI, USA.

### Bacterial infection, inhibitor treatment and human gastric mucosal biopsy specimen collection

*H. pylori* strains 26695, 8-1 and *H. felis* strain 49179 (#49179, ATCC, VA, USA) were cultured and maintained as reported previously.^52, 53^ Unless otherwise specified, GCCs were infected with strain 26695 at 200 MOI for the indicated period. To study proteasome-mediated degradation, cells were co-treated with the proteasome inhibitor MG132 for 6 h at 50 μM dose.

Endoscopic biopsy samples (from the antral region of the stomach) were collected from consenting patients who were suffering from stage III gastric adenocarcinoma following a National Institute of Science Education and Reseach (NISER) Review Board-approved protocol. Pathologically-confirmed gastric cancer cases with paired non-cancerous gastric tissues were included for the study. Patients with a history of previous *H. pylori* eradication therapy were excluded. The investigation was carried out in compliance with the Helsinki Declaration (2013) of the World Medical Association.

### Transient transfection and generation of stable cells

To transiently express Siah1, 1 × 10^6^ MKN45 cells were seeded in 6-well plates. On the next day, cells were transfected with 2.5 μg of plasmid DNA, 5 μl of P3000 reagent and 7.5 μl of Lipofectamine3000 reagent (Invitrogen). After 36 h, cells were infected with *H. pylori.* To knockdown expression of *ETS2 and siah1,* 0.2 × 10^6^ MKN45 cells were seeded in 6-well plates. On the next day, cells were transfected with 50 nM siRNA of ETS2 or Siah1 along with 10 μl of Lipofectamine3000 reagent (Invitrogen). To generate stable cell lines, MKN45 or AGS cells were seeded in 96 well plates 18–24 h before transfection. Stable transfectants were established with G418 selection.

### Real-time reverse transcription (RT)-PCR analysis

Total RNA was isolated from MKN45 cells after 30 min and 2 h of infection using an RNeasy kit (Qiagen, CA, USA). cDNA was synthesized and real-time RT-PCR was performed as described previously.^51^

### *In vitro* binding assay

Nuclear lysates were incubated with 5′ biotinylated double-stranded (ds) *siah1* EBS oligonucleotide (WT or Mut) coated on streptavidin-coated superparamagnetic beads (Dynabeads M-280 Streptavidin, Dynal, Invitrogen). Binding assays were performed as described previously.^51^ Oligos are shown in Supplementary Figure 1.

### Chromatin immunoprecipitation (ChlP) assay

ChIP assay was performed using QuikChIP chromatin immunoprecipitation kit (Imgenex, CA, USA) according to the manufacturer’s protocol.^51^ Chromatins were immunoprecipitated using ETS2 antibody (#22803, Santa Cruz Biotechnology, CA, USA). PCR amplification was performed to analyze the *in vivo* binding of ETS2 to *siah1* 5′ UTR EBS. Primers used for ChIP assay are shown in Supplementary Figure 1.

### Luciferase assay

Dual luciferase assays was performed to study activity of *siah1* promoter after *H. pylori* infection. Cells were co-transfected either with the WT or ETS2-Mut *siah1* luciferase constructs (cloned in pGL3 basic vector) and the phRLTK *Renilla* luciferase constructs at a ratio of 50:1 using Lipofectamine 2000 reagent (Invitrogen). For another set of experiment, cells were co-transfected with WT *siah1* luciferase construct along with ETS2 overexpression plasmid and phRLTK *Renilla* luciferase construct at a ratio of 25:25:1 using Lipofectamine 2000 reagent (Invitrogen). At 36 h of transfection, uninfected or 2 h *H. pylori*-infected cells were lysed with passive lysis buffer (Promega) and analyzed for luciferase activity as described earlier.^51^

### Immunoblotting and antibodies

Nuclear, cytoplasmic and membrane proteins were isolated from *H. pylori*-infected MKN45 cells using NE-PER Nuclear and Cytoplasmic Extraction Reagents (Pierce, Rockford, IL, USA). Around 2 × 10^6^ MKN45 cells were seeded in 60 mm cell culture dish 24 h before infection. Postinfection, cytoplasmic and nuclear fractions were isolated as per manufacturer’s instruction, whereas membrane proteins were isolated from the cytoplasmic extract after further centrifugation at 16000*g* for 45 min at 4 °C. Whole cell extracts were prepared from uninfected or *H. pylori*-infected GCCs. For immunoblotting, proteins were resolved on SDS-PAGE gel and blotted onto PVDF membranes. The following primary antibodies were used: Siah1 (1:250) (#300974, Novus Biologicals, CO, USA), ETS2 (1:1000) (#22803, Santa Cruz Biotechnology, CA, USA), E-cadherin (1:1000) (#3195, Cell Signalling Technology, MA, USA) and β-catenin (1:5000) (#32572, Abcam, MA, USA). α-tubulin (1:5000) (#52866, Abcam), histone-deacetylase 1 (HDAC1) (1:1000) (#2062, Cell Signalling Technology), Pan-cadherin (1:1000) (#4068, Cell Signalling Technology) antibodies were used for normalization of protein loading. Immunoreactive bands were detected as described earlier.^51^

### Coimmunoprecipitation assay

For, coimmunoprecipitation assays, 5 × 10^6^ MKN45 cells were seeded in 100 mm cell culture dish 24 h before infection. Membrane fractions were isolated and were incubated with Siah1 primary antibody (Novus) for overnight at 4 °C. The protein-antibody complex was pulled down using 50% A/G agarose followed by washing with ice-cold phosphate-buffered saline (PBS). Proteins were denatured by using Laemmli buffer (HiMedia, Mumbai, India) and analyzed by western blotting.

### Cell migration and invasion assays

AGS cells were transfected with or control duplex RNA and siRNA of Siah1. Cell migration and invasion assay was performed following a previously-described protocol.^51^

### Wound-healing assay

To study the effect of Siah1 on the wound-healing property of GCCs, adherent AGS cells were preferred than semi-adherent MKN45 cells. AGS cells stably-expressing *siah1* or pcDNA3.1^+^ were seeded and wound-healing assay was performed as described previously.^51^

### Soft agar assay

Anchorage-independent growth was studied by performing soft-agar assay using the empty vector (pcDNA3.1^+^) or ETS2 or Siah1-expressing MKN45 stable cells following a previously-described method.^51^

### *H. felis* infection in C57BL/6 mice

Four to five week-old both male and female C57BL/6 mice were procured from the National Centre for Laboratory Animal Sciences of the National Institute of Nutrition (Hyderabad, India). The study was performed after getting the institutional animal ethics committee (IAEC) approval provided by NISER (approval No SBS-AH/03/13/05). Male and female mice of 7–8 weeks of age were randomly and blindly divided into two groups- uninfected and infected (16 mice in each group) and infected with *H. felis* as mentioned earlier.^25^ After 8 months of observation, mice were killed and the stomach was isolated from uninfected and infected animals. After fixation, antral sections (5 μm) were either stained with H&E or processed for immunofluorescence microscopy as mentioned below.

### Confocal and immunofluorescence microscopy and H&E staining

MKN45 cells were seeded on glass coverslips 24 h before infection to study endogenous levels of various proteins. For transfection-based assays, MKN45 cells were seeded on coverslips 24 h before transfection and were transfected using Lipofectamine3000 (Invitrogen). Nontransfected and transfected cells were either infected with *H. pylori* for 20 h or left uninfected followed by fixation and staining.^51^ Cells were incubated with Siah1 (1:100, #sc101252, Santa Cruz Biotechnology) or β-catenin (1:250, Abcam) or E-cadherin (1:100, Cell Signalling Technology) primary antibodies for overnight and Images were taken using a confocal microscope.^51^

For immunostaining, 5 μm thick gastric adenocarcinoma biopsy specimen were fixed as before.^51^ Fixed sections were incubated with primary antibodies against Siah1 (1:50, #ab49177, Abcam) or ETS2 (1:100, Santa Cruz Biotechnology) followed by incubation with secondary antibodies. Images were taken following a previously-described method.^51^ H&E staining was done following standard procedure.

### Statistical analysis

*In vitro* experiments had a minimum of three independent experiments, each with three technical replicates. No statistical method was used to predetermine the sample size. Experiments were performed and analyzed in non-randomized and non-blinded fashion (except for animal studies). Statistical analysis of data was performed by Student’s *t*-test for comparisons involving two groups. Values were given as mean±s.e.m. Statistical significance was determined at *P<0.05. Two-way ANOVA was performed to compare various transfection groups. Tukey’s test was done for post hoc comparisons. There was no estimate of variation within each group.

## Abbreviations

ChIP: Chromatin immunoprecipitation
*cag*: Cytotoxin-associated gene
DAPI: 4′, 6-Diamidino-2-Phenylindole, Dilactate
EBS: E26 transformation-specific sequence 2-binding site
ETS2: E26 transformation-specific sequence 2
GCCs: Gastric cancer cells
H&E: Haematoxylin and eosin; HDAC1, Histone-deacetylase 1
*H. pylori*: *Helicobacter pylori*
MOI: Multiplicity of infection
MDM2: Mouse double minute 2 homolog
PAI: Pathogenicity island
PBS: Phosphate-buffered saline
PVDF: Polyvinylidene fluoride
Real-time RT-PCR: Real-time reverse transcription PCR
RING: Really interesting new gene
Siah: The seven in absentia homolog
SIP: Siah-interacting protein
