# Supplementary material for: Membrane-bound β-catenin degradation is enhanced by ETS2-mediated Siah1 induction in Helicobacter pylori-infected gastric cancer cells
Source: Oncogenesis. 2017 May 8;6(5):e327–. doi: 10.1038/oncsis.2017.26 (PMC5523059; doi:10.1038/oncsis.2017.26)
Supplement: Supplementary Information [file oncsis201726x1.pdf]

Supplementary data

Membrane-bound β-catenin degradation is enhanced by ETS2-mediated Siah1 induction in *Helicobacter pylori*-infected gastric cancer cells

L Das[1,5], SB Kokate[1,5], P Dixit[1], S Rath[1], N Rout[2], SP Singh[3], SE Crowe[4], A Bhattacharyya[1]

Affiliations: [1]School of Biological Sciences, National Institute of Science Education and Research (NISER) Bhubaneswar, HBNI,P.O. Bhimpur-Padanpur, Via Jatni, Dist. Khurda 752050, Odisha, India. [2]Department of Oncopathology, Acharya Harihar Regional Cancer Centre, Cuttack 753007, Odisha, India. [3]Department of Gastroenterology, SCB Medical College, Cuttack 753007, Odisha, India. [4]School of Medicine, Division of Gastroenterology, UC San Diego, California 92093, USA

[5]contributed equally

Correspondence: School of Biological Sciences, National Institute of Science Education and Research (NISER) Bhubaneswar, HBNI, P.O. Bhimpur-Padanpur, Via Jatni, Dist. Khurda 752050, Odisha, India. Tel: +91-674-2494210, e-mail: asima@niser.ac.in

siah1 primers

***siah1* forward:** 5′AAAAGCTTCAAAATGAGCCGTCAGACTGCTACAGCATTA 3′
***siah1* reverse:** 5′CCTCGAGTCAACACATGGAAATAGTTACATTGATG 3′

siah1 5’ UTR primers

WT forward: 5′AAGGTACCAGCAACGGTAGC 3′
WT reverse: 5′ATTAAGCTTTGTGGGCGGAGAG 3′

EBS mutant: 5′ GTCGGTCTCGGCGCC***ATT***AAGAGGCGGTGGCGC 3′

siah1 5’ UTR EBS oligos for *in vitro* binding assay

5’ biotinylated WT forward: 5′ TCTCGGCGCCGGGAAGAGGCGGTGGCG 3′
5’ biotinylated WT reverse: 5′ CGCCACCGCCTCTTCCCGGCGCCGAGA 3′

5’ biotinylated mutant forward: 5′TCTCGGCGCC***ATT***AAGAGGCGGTGGCG 3′
5’ biotinylated mutant reverse: 5′CGCCACCGCCTCTTAATGGCGCCGAGA 3′

Primers for ChIP assay

Forward (Specific): 5′ CGCAGTGTGTGGTATTTAGCG 3′
Reverse (Specific): 5′ TCCTGGCACCAACGCG 3′

Forward (Non Specific): 5′ AACACTAGCCACTACTTGAGGTTTA 3′
Reverse (Non Specific): 5′ GCAGCTTTTGATGGTGGCTTTAC 3′

Supplementary Figure 1. List of primers and oligonucleotides used in the study. Underlined is the EBS. Mutations are shown in boldfaced italics.

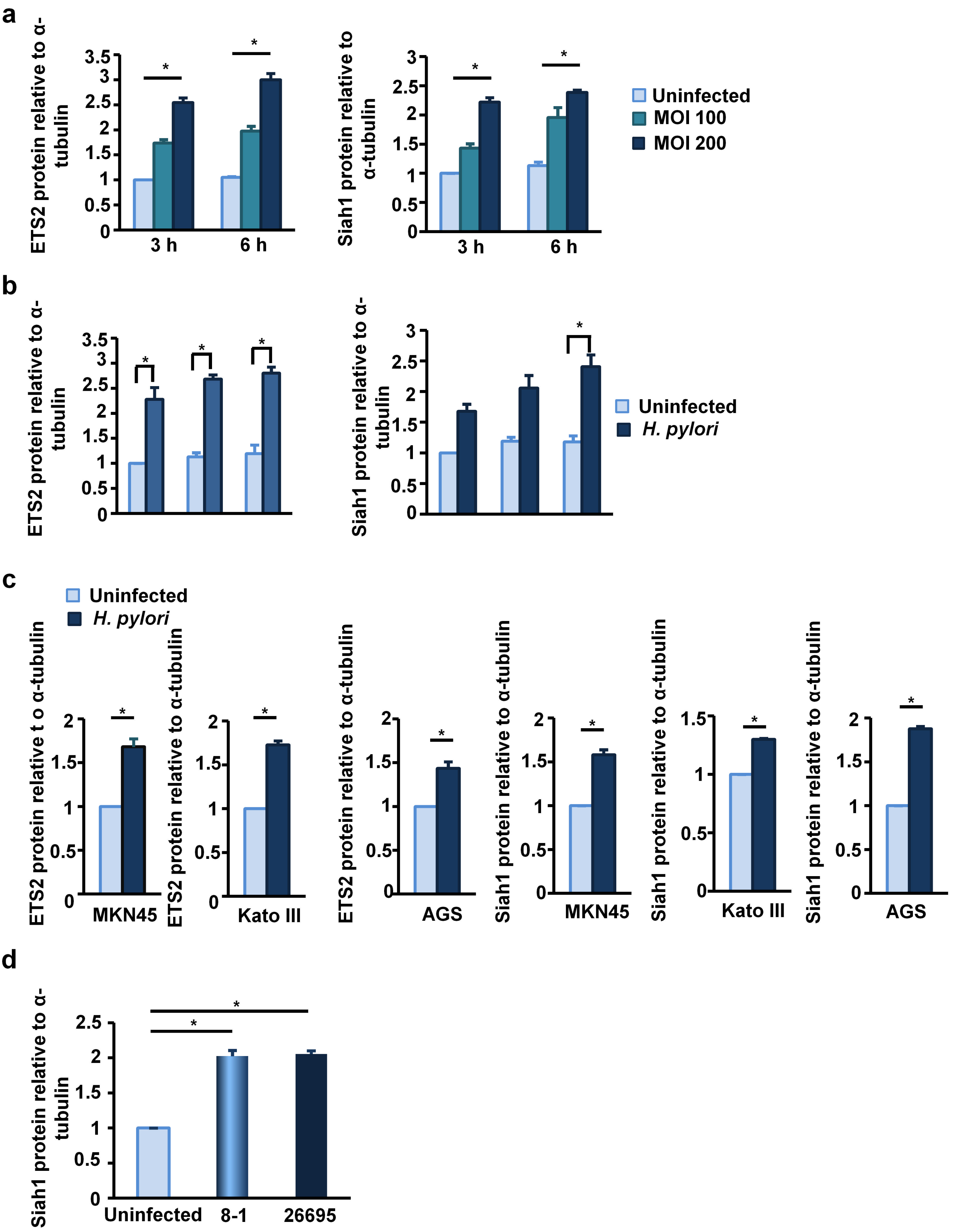

Supplementary Figure 2. Induced ETS2 and Siah1 expression in *H. pylori*-infected GCCs. Graphical representations of Fig. **3a**-**3d** (mean ± SEM, n=3) *$P < 0.05$.

Protein level relative to loading control (Expressed as Protein/loading control)

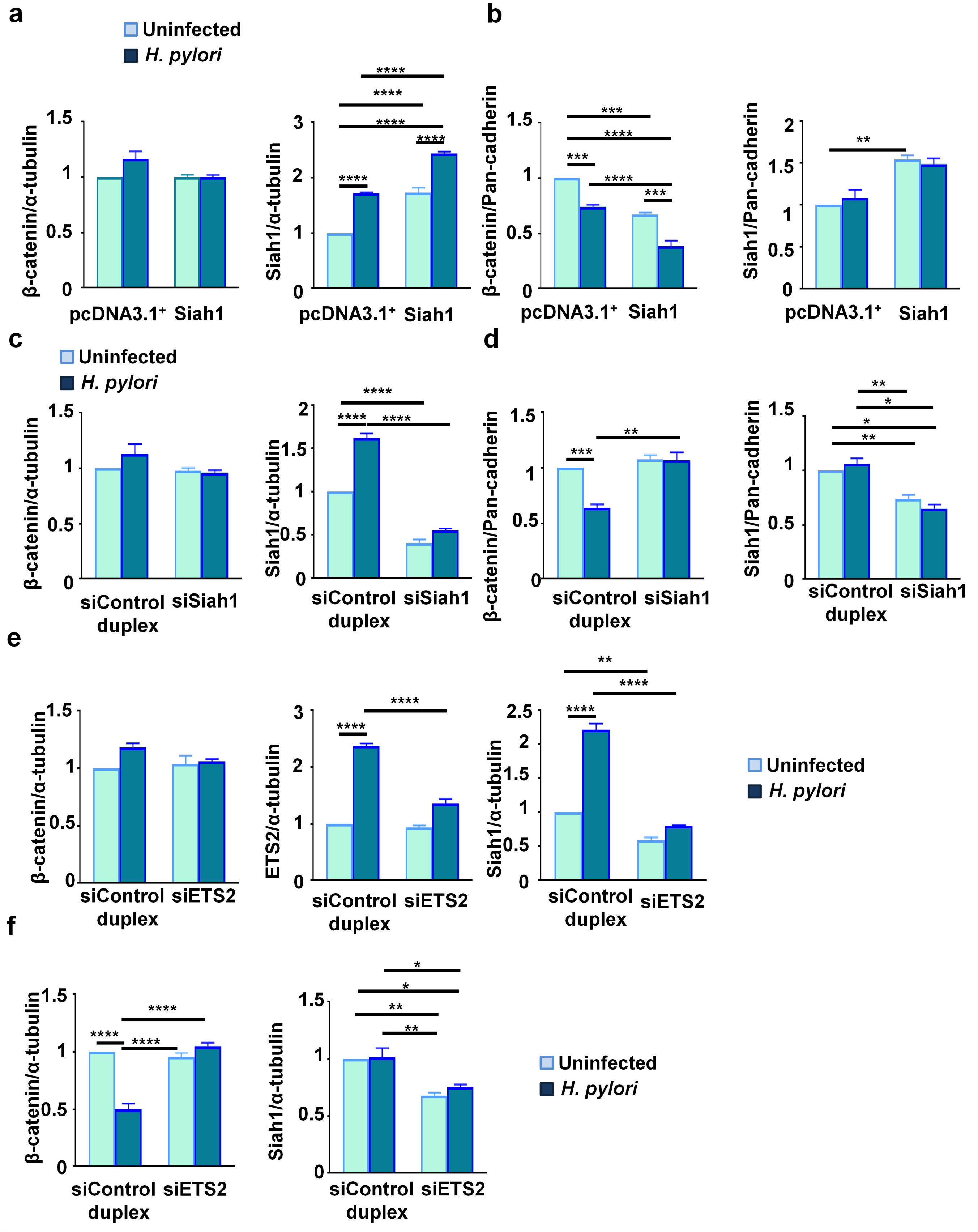

Supplementary Figure 3. Increased membrane-bound β-catenin degradation in *H. pylori*-infected GCCs is mediated by Siah1. Graphical representations of Fig. **6a-f** (n=3). Data are analyzed by 2-way ANOVA with Tukey's post hoc test. Error bars, SEM. **P* < 0.05; ***P* < 0.01; ****P* < 0.003; *****P* < 0.0001.

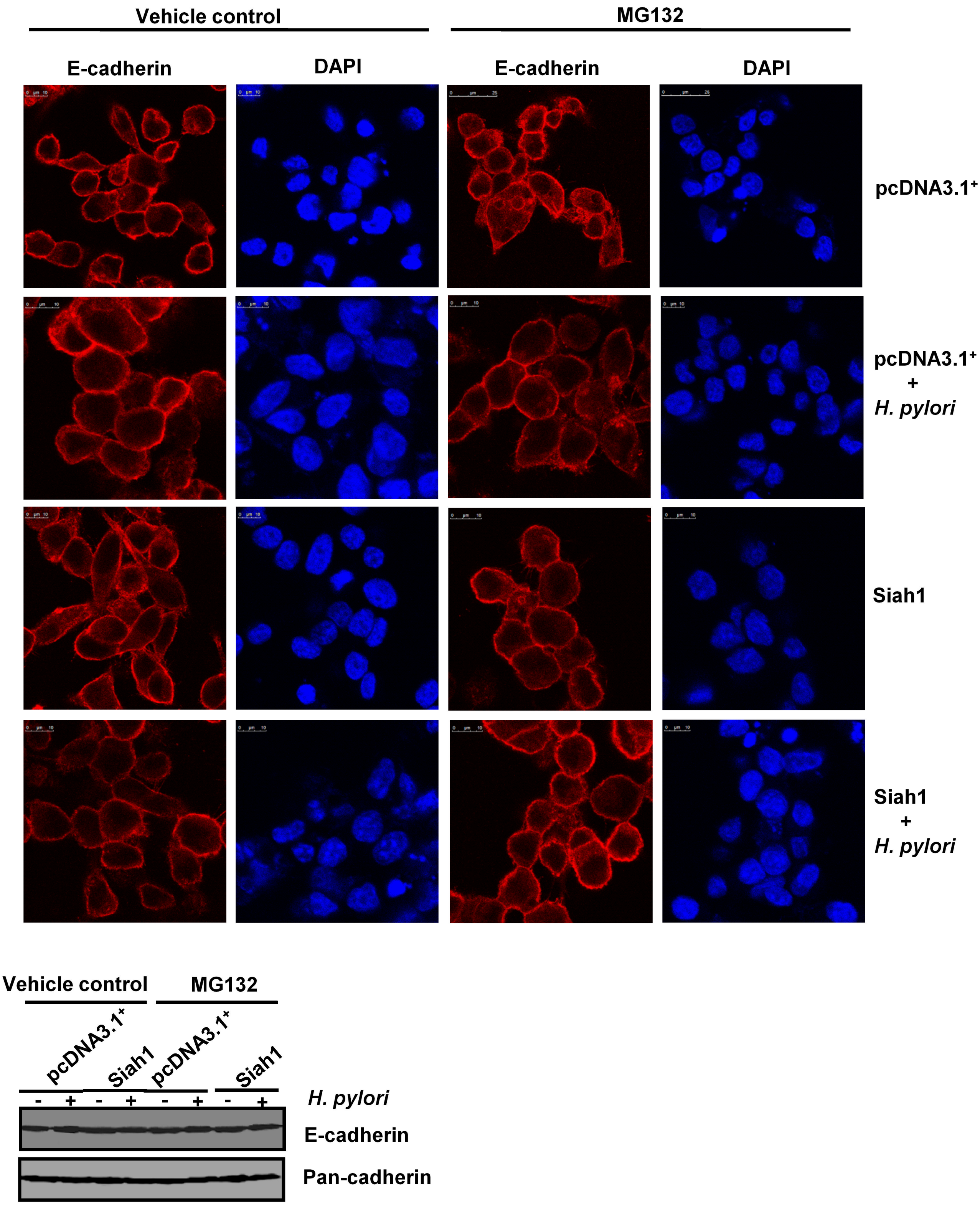

Supplementary Figure 4. The status of Membrane bound E-cadherin in *H. pylori* infected GCCs. A representative confocal microscopy result (n=4) showing E-cadherin expression in *H. pylori*-infected MKN45 cells. Accompanying western blot result showing level of E-cadherin in the above experiment.
